# The Molecular Signature of Early-Onset Colorectal Cancer Liver Metastases: Distinct Biology and Clinical Challenges

**DOI:** 10.3390/ijms27073289

**Published:** 2026-04-04

**Authors:** Sophia Tsokkou, Ioannis Konstantinidis, Paraskevi Chatzikomnitsa, Menelaos Papakonstantinou, Areti Danai Gkaitatzi, Evdokia Toutziari, Dimitrios Alexandrou, Dimitrios Giakoustidis, Vasileios N. Papadopoulos, Alexandros Giakoustidis

**Affiliations:** 1 First Department of Surgery, General Hospital Papageorgiou, Aristotle University of Thessaloniki, 56429 Thessaloniki, Greece; stsokkou@auth.gr (S.T.); voula.hatzikomnitsa@yahoo.gr (P.C.); menelaospap.md@gmail.com (M.P.); aretidanaegtz24@gmail.com (A.D.G.); evdo-t@hotmail.com (E.T.); alexandrousurgeon@gmail.com (D.A.); dgiak@auth.gr (D.G.); papadvas@auth.gr (V.N.P.); 2 Laboratory of Histology-Embryology, Department of Medicine, Faculty of Health Sciences, Aristotle University of Thessaloniki, 54124 Thessaloniki, Greece

**Keywords:** early-onset colorectal cancer, liver metastases, genomic instability, copy number variations, immune microenvironment, ferroptosis, *PIK3CA*-mutant CRC, immunotherapy resistance, molecular profiling, precision oncology

## Abstract

Early-onset colorectal cancer (EOCRC), defined as diagnosis before the age of 50 years, is increasing globally and is frequently characterized by aggressive biology and a disproportionate burden of liver metastases. This review synthesizes emerging evidence on the distinct molecular, immunologic and clinical features that differentiate EOCRC liver metastases from those arising in older adults. Genomic studies revealed increased chromosomal instability, increased copy number variation burden and unique amplification patterns involving *MYC*, *RAD21*, *GNAS* and *MAPK1*, alongside altered frequencies of classical driver mutations and increased germline predisposition. EOCRC liver metastases also exhibit a progenitor-like transcriptional state and an immune-cold microenvironment marked by reduced myeloid infiltration, impaired antigen presentation and profound resistance to immunotherapy, particularly in microsatellite-stable disease. Mechanistic insights into ferroptosis highlight therapeutic vulnerabilities, especially in *PIK3CA*-mutant tumors, where aspirin and ferroptosis inducers show synergistic potential. Clinically, high-risk EOCRC patients often present with left-sided primary tumors, synchronous metastases, adverse histology, elevated CEA levels and a hereditary predisposition, with prognostic models incorporating these variables outperforming traditional staging. Collectively, accumulating evidence suggests that EOCRC liver metastases may represent a biologically and clinically distinct entity, although ongoing debates regarding the extent of this distinction underscore the need for age-specific molecular profiling and prospectively validated therapeutic strategies.

## 1. Introduction

Colorectal cancer (CRC) has traditionally been perceived as a condition that predominantly impacts older individuals. However, its epidemiological profile has undergone significant changes in the last twenty years. The prevalence of EOCRC, characterized as CRC detected before the age of 50 years, is increasing at a concerning rate globally, necessitating an urgent comprehension of its fundamental biology and clinical characteristics. EOCRC accounts for 10% of all colorectal cancer cases, and its incidence is increasing, particularly in high-income countries. Patients often present with advanced disease in the left colon, and one in six patients has deficient DNA mismatch repair [1,2,3]. One of the most severe manifestations of EOCRC is the emergence of liver metastases, which continue to be the primary cause of CRC-related mortality. Liver metastases represent the single greatest determinant of survival in patients with CRC. As the liver is the primary site of hepatic clearance of venous blood components from the colon, it is the most common site of metastatic spread. In EOCRC, this pattern is not only preserved but also often more aggressive, creating a disproportionate clinical impact. Recent evidence indicates that EOCRC liver metastases are not simply early manifestations of a typically late-onset illness but may instead constitute a biologically different entity characterized by unique molecular causes, metastatic patterns and treatment vulnerabilities [4,5,6,7,8].

Despite advancements in genomic profiling and precision oncology, the molecular landscape of early-onset colorectal cancer liver metastases remains poorly defined. Preliminary research suggests that these tumors may possess a distinct array of genetic modifications, characteristics of the immunological milieu and activation of signaling pathways in contrast to those that manifest later in life. These disparities may lead to a more aggressive clinical trajectory frequently observed in younger patients, characterized by fast metastatic advancement, resistance to standard treatments and inferior overall prognoses. Comprehending these molecular fingerprints is crucial for clarifying disease causes, guiding personalized therapy approaches and enhancing prognosis accuracy [6,8,9].

The present review aims to explore the distinct molecular and clinical features of EOCRC liver metastases, highlighting emerging biological insights and the challenges they pose for current management paradigms. By integrating recent advances in tumor genomics, transcriptomics and microenvironmental analysis, we aim to clarify how EOCRC metastases differ from those arising in older adults and to highlight the need for age-specific approaches in both research and clinical practice.

## 2. Molecular and Biological Features

### 2.1. Distinct Biology of Early-Onset Colorectal Cancer

**Figure 1:**
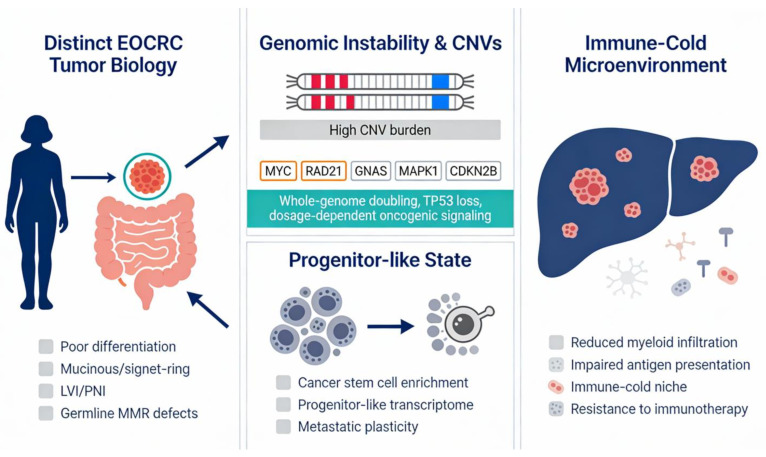
Molecular and biological features of early-onset colorectal cancer liver metastases. Schematic overview illustrating (1) distinct EOCRC tumor biology in young patients with aggressive clinicopathologic features and a germline predisposition, (2) high chromosomal instability and copy number variation burden with amplifications in *MYC*, *RAD21*, *GNAS*, and *MAPK1* and loss of *CDKN2B*, (3) a progenitor-like, stem-cell-enriched transcriptional state and (4) the development of an immune-cold liver metastatic niche characterized by reduced myeloid infiltration, impaired antigen presentation and resistance to immunotherapy. The figure is organized as four interconnected modules, with arrows between modules indicating mechanistic relationships. EOCRC, early-onset colorectal cancer; LOCRC, late-onset colorectal cancer; CNV, copy number variation; TME, tumor microenvironment; CIN, chromosomal instability; dMMR, deficient DNA mismatch repair.

Growing evidence indicates that EOCRC represents a biologically distinct entity rather than a younger presentation of traditional late-onset colorectal cancer (LOCRC). Thorough reviews highlight persistent clinicopathologic disparities, notably a greater incidence of poorly differentiated, mucinous and signet ring cell histology, alongside elevated rates of lymphovascular and perineural invasion, all of which are indicative of more aggressive tumor behavior in younger adults. Molecular investigations provide additional corroboration for this differentiation: EOCRC tumors frequently exhibit a modified frequency of classical driver mutations, including variations in *APC*, *KRAS* and *TP53* mutation patterns, indicating divergent carcinogenic pathways relative to older patients. Systematic evaluations indicate specific epigenetic and transcriptome characteristics, encompassing discrete DNA methylation profiles and pathway activation, hence validating the notion of differential tumor progression in EOCRC. Moreover, EOCRC is characterized by an abundance of germline pathogenic mutations, especially abnormalities in mismatch repair genes linked to Lynch syndrome, highlighting a more pronounced genetic factor in younger individuals. The combination of clinicopathologic, genomic and epigenetic abnormalities offers substantial evidence that EOCRC has a distinct biological basis, significantly influencing metastasis, treatment efficacy and prognosis [10,11,12,13] (Figure 1).

### 2.2. Copy Number Variations (CNVs) and Genomic Instability

Recent single-cell integration analyses, including the study by Peng and Zhan, revealed that, compared with LOCRC, EOCRC has an increased burden of CNVs. This enhanced genomic instability is a unique molecular feature of EOCRC and is correlated with more advanced TNM stages at diagnosis, greater tumor heterogeneity and resistance to conventional chemotherapy, as well as increased metastatic potential [14]. This CNV burden in EOCRC is also confirmed by genomic profiling studies, which have shown that chromosomal instability and whole-genome doubling events, often driven by early TP53 loss, are more prevalent in EOCRC than in LOCRC, contributing to aggressive clinical behavior [15]. Furthermore, specific patterns of CNVs have been identified, such as genes with copy number gains that are located primarily on chromosomes 1, 16, 19 and 21, whereas copy number losses are more frequently found on chromosome 6 [14,16].

These distinct genomic variations help distinguish EOCRC from LOCRC and may cause the accelerated clinical progression and poorer prognosis observed in younger patients. Clinically, the need for personalized therapeutic strategies and molecular profiling should guide management, as recommended by the Delphi Initiative for EOCRC International Management Guidelines and the National Comprehensive Cancer Network, which advocate for universal germline and somatic genomic testing in EOCRC [4].

### 2.3. The Molecular Paradox of EOCRC: Similar Drivers, Distinct Profiles

Compared with that of LOCRC, the molecular signature of EOCRC liver metastasis is characterized by distinct genomic and transcriptomic profiles defined by significant nuances in clonal evolution. While both cohorts share a common ‘trunk’ of mutations, EOCRC liver metastases exhibit a relatively high frequency of amplification of genes such as *MYC*, *RAD21*, *GNAS* and *MAPK1*. These amplifications, particularly at the 8q24 locus, suggest that EOCRC utilizes dosage-dependent signaling, which drives malignancy rather than relying solely on novel point mutations [17,18,19,20]. Furthermore, compared with LOCRC, recurrent mutations in *APC*, *NRAS Q61L*, *PIK3CA* and *TP53* G266V are associated with a poorer prognosis, likely due to their role in enhancing metabolic plasticity and resisting ferroptosis [17,18]. A defining feature of the EOCRC metastasis niche is the loss of *CDKN2B*. This alteration, alongside the aforementioned amplifications, suggests profound activation of oncogenic pathways related to cell proliferation and the stress response, contributing to more aggressive biological phenotypes [17,21,22]. Paradoxically, large multi-institutional analyses have shown that mutation rates in key driver genes (*KRAS*, *BRAF*, *APC*, *TP53*, *PIK3CA*, *SMAD4*) are comparable between early- and late-onset colorectal liver metastases, indicating that age at onset alone does not confer a unique mutational landscape in metastatic lesions and that outcomes after hepatectomy are similar across age groups [23,24]. However, the clinical reality of EOCRC is characterized by increased virulence. Patients under 50 years of age tend to present with more aggressive clinicopathological features, including higher rates of synchronous metastasis, distal-rectal primaries and extensive lymph node involvement. Recent transcriptomic evidence further suggests that EOCRC metastasis often occurs in a ‘progenitor-like’ state characterized by an enrichment of cancer stem cell markers and an immune-cold microenvironment, which may explain the rapid progression observed in younger populations despite the similarities in their primary genetic drivers [1,19,23].

### 2.4. Immune Microenvironment and Immunogenicity

Compared with LOCRC, EOCRC has a distinctly different immune microenvironment characterized by reduced infiltration of myeloid cells, including dendritic cells and macrophages. Single-cell integration analyses have proven this reduced myeloid cell presence in EOCRC, which adds to an “immune-cold” phenotype particularly evident in liver metastases [14]. However, this immune landscape of EOCRC is paradoxical, while it shows reduced immune cell infiltration, it is associated with increased tumor mutational burden (TMB) and increased PD-L1 expression, alongside immune-related signaling pathways [15,16].

The paradox described above is borne out across multiple studies. Single-cell RNA sequencing has confirmed that EOCRC tumors harbor a more immunosuppressive TME than does late-onset CRC (LOCRC), with downregulated interferon-gamma responses in effector T cells, stronger TGF-β signaling in regulatory T cells, and reduced antigen presentation by dendritic cells and macrophages [4,25]. A 2025 ASCO analysis of TCGA data similarly revealed decreased CD47 expression and increased BTLA expression in EOCRC, both of which may impair immune cell infiltration and CD8+ T-cell function [26]. Transcriptomic analyses of a New Zealand cohort revealed that 94% of EOCRC tumors were classified as immune-depleted (CMS2/CMS3) tumors, whereas 67% of LOCRC tumors were classified as immune-depleted [27].

However, the picture is not uniformly immunosuppressive. A 2025 peripheral blood study demonstrated that EOCRC patients exhibit a heightened proinflammatory peripheral environment with increased CD4+ Th1, Th9, and Th17 functional capacity and elevated plasma IFN-γ and CXCL8/IL-8, whereas LOCRC patients presented hallmarks of immunosenescence [28]. Importantly, EOCRC tumors also display greater T-cell receptor (TCR) diversity than AOCRC tumors do, suggesting a more diverse intratumoral T-cell response that is nonetheless functionally constrained [29]. A Swedish study using multiplex immunofluorescence and NanoString profiling of 770 tumor immunity genes revealed no significant global differences in T-cell infiltration or inflammatory mediators between matched EOCRC and AOCRC tumors, cautioning against overgeneralization [30].

These seemingly contradictory findings may be reconciled by the distinction between systemic immune activation (preserved or even enhanced in EOCRC) and local tumor-immune interactions (suppressed). The microbiome data add another layer: EOCRC tumors present distinct microbial signatures with positive correlations between intratumoral microbes and regulatory T cells and accelerated epigenetic aging (methylation ages ~15 years older than chronological age), suggesting that environmental exposure may drive both immune dysregulation and tumorigenesis [31,32].

Moreover, the kinase LMTK3 has been identified as a potential biomarker for EOCRC. Further multiomics analysis revealed *LRRC26* and *REP15* as novel prognosis-related driver genes in CRC, alongside the identification of 66 putative susceptibility genes, including *DIP2B* and *SFMBT1* [33,34]. The downregulation of ligands for immune cell recruitment in EOCRC tumor cells is linked to reduced tumor–immune interactions, suggesting impaired paracrine signaling that normally drives immune cell recruitment and activation [14].

Liver metastases from colorectal cancer profoundly reduce immunotherapy efficacy through multiple mechanisms, even in microsatellite instability-high (MSI-H)/mismatch repair deficient (dMMR) disease. Yu et al. demonstrated in preclinical models that liver metastases siphon activated CD8+ T cells from systemic circulation, where FasL+CD11b+F4/80+ hepatic macrophages induce the apoptosis of antigen-specific Fas+CD8+ T cells, creating a “systemic immune desert” [35]. Clinically, the CCTG CO.26 secondary analysis confirmed that in microsatellite-stable (MSS) CRC patients treated with durvalumab plus tremelimumab, patients without liver metastases had a DCR of 49% versus 14% in those with liver metastases, with significantly improved PFS (HR 0.54). Wang et al. reported that in MSS CRC patients treated with PD-1/PD-L1 inhibitors, patients without liver metastases achieved an ORR of 19.5% versus 0% in those with liver metastases, with liver metastasis being the most significant independent predictor of poor PFS in multivariate analysis (HR 7.00) [36,37].

Several approaches are being investigated to overcome this resistance. The METIMOX trial demonstrated that alternating short-course oxaliplatin-based chemotherapy with nivolumab significantly improved PFS in MSS CRC patients >60 years with abdominal metastases, with 17% achieving durable complete responses [38]. A post hoc analysis revealed that this regimen was particularly effective for liver and lymph node metastases from right-sided tumors with intermediate TMB or BRAF mutations but ineffective for peritoneal and lung metastases [39].

Emerging approaches also include combining immune checkpoint inhibitors with anti-VEGF agents plus chemotherapy, which has shown promising results, with an ORR of 42.4% and a DCR of 92% in some studies, particularly when used as first-line therapy [40,41]. Other strategies under investigation include combinations of MEK inhibitors, TGF-β pathway inhibitors, EGFR inhibitors and liver-directed radiotherapy to eliminate immunosuppressive hepatic macrophages and restore systemic antitumor immunity [35,40,41,42,43,44]. The combination of regorafenib, a multikinase inhibitor with antiangiogenic properties, plus immune checkpoint inhibitors has also demonstrated encouraging activity in MSS CRC, with improved outcomes in patients with RAS/RAF wild-type tumors [45,46].

Furthermore, additional suppressive mechanisms in the liver include a paucity of dendritic cells, Treg-derived IL-10, the TGF-β dual barrier, PGE2-driven terminal exhaustion, and Prok2^+^ neutrophils [47,48]. Dendritic cell paucity was supported by Ho et al. (2021), who demonstrated that, compared with subcutaneous tumors, pMMR CRC liver metastases have a marked deficiency in both activated T cells and dendritic cells and that Flt3L supplementation could increase dendritic cell infiltration and improve survival when combined with ICB [48]. Treg-derived IL-10 was supported by Shiri et al. (2024), who reported that Foxp3^+^ Tregs are the major source of IL-10 in liver metastatic sites, acting in an autocrine loop to amplify IL-10 production and upregulate PD-L1 on monocytes, thereby attenuating CD8^+^ T-cell cytotoxicity [49]. The TGF-β dual barrier was supported by Henriques et al. (2025), who demonstrated that TGF-β simultaneously blocks peripheral memory CD8^+^ T-cell recruitment and instructs SPP1^+^ macrophages to suppress clonal T-cell expansion through collagen deposition, coordinating immunosuppression across innate and adaptive compartments [50]. PGE2-driven terminal exhaustion was supported by Fumet et al. [51], who reported that M2-like tumor-associated macrophages in MSS CRC produce PGE2 via COX1/2, driving PD-1^+^TIGIT^+^ terminal exhaustion in CD8^+^ T cells with minimal cytokine secretion. Notably, COX2 inhibition or TIGIT blockade restored CD8^+^ T-cell function and improved anti-PD-L1 efficacy [51]. Finally, Prok2^+^ neutrophils were supported by Jiang et al. (2025), who used single-cell transcriptomics to identify a Prok2^+^ neutrophil subpopulation with high PD-L1 expression in the liver metastatic niche that suppresses macrophage phagocytosis and promotes T-cell exhaustion [52].

### 2.5. Targeting Ferroptosis: Mechanistic Basis for Therapeutic Intervention

Ferroptosis is an iron-dependent form of programmed cell death characterized by the accumulation of lipid peroxides, distinct from apoptosis, necrosis and autophagy. This regulated cell death mechanism has emerged as a promising therapeutic strategy for CRC, particularly in overcoming resistance to conventional therapies and enhancing immunotherapy efficacy [53,54,55].

While ferroptosis has emerged as a broadly relevant mechanism in CRC biology, its importance in the context of EOCRC liver metastasis is particularly compelling and warrants specific attention. EOCRC tumors are characterized by a high prevalence of *PIK3CA* mutations, altered lipid metabolism and an immune-cold microenvironment in liver metastases, three features that converge directly on ferroptosis susceptibility and resistance pathways. The *PIK3CA*-mutant subgroup, which is disproportionately represented in EOCRC, activates the PI3K/AKT/mTOR axis to upregulate SCD1-mediated monounsaturated fatty acid synthesis, thereby suppressing ferroptosis as a survival mechanism that is specifically relevant to the metabolic milieu of the hepatic metastatic niche. Furthermore, the capacity of ferroptosis induction to reshape the immune microenvironment from cold to hot is of particular therapeutic interest in EOCRC, where conventional immunotherapy is limited and the liver microenvironment is profoundly immunosuppressive. The subsections that follow describe the mechanistic basis of ferroptosis in CRC, with specific attention given to these EOCRC-relevant features.

#### 2.5.1. Molecular Mechanisms of Ferroptosis in Colorectal Cancer

The regulation of ferroptosis in CRC involves multiple interconnected molecular pathways. Glutathione peroxidase 4 (GPX4) serves as the master negative regulator of ferroptosis, catalyzing the reduction in lipid hydroperoxides using glutathione (GSH) as a cofactor [54,55,56]. The inhibition of GPX4 with compounds such as RSL3 triggers potent ferroptosis in CRC cells [54,55]. System Xc (the cystine/glutamate antiporter composed of *SLC7A11* and *SLC3A2*) is critical for the import of cystine, which is subsequently converted to cysteine for GSH synthesis. Erastin and other ferroptosis inducers block System Xc, depleting cellular cysteine and triggering ferroptosis [54,55].

Additional ferroptosis regulators in CRC include NCOA4 (nuclear receptor coactivator 4), which mediates ferritinophagy to increase the labile iron pool and increase ferroptosis sensitivity, and the PERK-Nrf2-HO-1 pathway, which can promote ferroptosis through alternations in iron metabolism and antioxidant systems [56,57,58]. The transcription factor Nrf2 functions as a ferroptosis gatekeeper by upregulating *GPX4*, *SLC7A11*, ferritin heavy chain (FTH1) and ferroportin (FPN), thereby suppressing ferroptosis [57].

#### 2.5.2. Ferroptosis and *PIK3CA*-Mutant Colorectal Cancer

A particularly compelling therapeutic opportunity exists in *PIK3CA*-mutant CRC, where aspirin has demonstrated significant clinical benefit. Some studies reported modestly higher *PIK3CA* mutation rates in EOCRC patients than in LOCRC patients, such as 20% vs. 12.1% in a Thai cohort and an OR of 1.24 (*p* = 0.041) in a Chinese cohort of 4468 patients, particularly those with left-sided tumors [59,60]. In the TMB-high/MSI-H subgroup, *PIK3CA* H1047R was significantly enriched in EOCRC (22% vs. 9%, *p* < 0.01), and PI3K pathway mutations were more common in TMB-high/MSS EOCRC (74% vs. 56%, *p* < 0.01) [61]. However, it should be noted that a large 2020 US cohort reported a lower frequency of *PIK3CA* mutations in EOCRC patients (age <40) than in age-related CRC patients [62].

The landmark ALASCCA trial demonstrated that 160 mg daily aspirin led to a significantly lower 3-year cumulative incidence of recurrence (7.7% vs. 14.1%, HR 0.49, 95% CI 0.24–0.98, *p* = 0.04) in patients with resected CRC harboring *PIK3CA* hotspot mutations in exons 9 or 20. Similar benefits were observed in patients with other somatic alterations in *PIK3CA*, *PIK3R1* or PTEN (7.7% vs. 16.8% recurrence, HR 0.42) [63]. These findings were corroborated by the SAKK 41/13 trial, which revealed a trend toward improved disease-free survival with aspirin in *PIK3CA*-mutant colon cancer patients (HR 0.57, 90% CI 0.27–1.22) [64].

The mechanism underlying the efficacy of aspirin in *PIK3CA*-mutant CRC involves the inhibition of COX-2-mediated PGE2 production, which disrupts the signaling axis involving COX-2, PGE2, and the PI3K pathway [63,65]. Preclinical studies have demonstrated that aspirin combined with ferroptosis inducers such as RSL3 effectively induces ferroptosis in *PIK3CA*-mutant CRC cells by inhibiting the mTOR/SREBP-1/SCD1 signaling pathway, which controls lipogenic metabolism [66]. The PI3K/AKT/mTOR pathway, which is hyperactivated by *PIK3CA* mutations, normally promotes cell survival and suppresses ferroptosis through *SREBP1*-mediated induction of stearoyl-CoA desaturase-1 (SCD1), which produces monounsaturated fatty acids that protect against lipid peroxidation. Aspirin-mediated mTOR inhibition synergizes with RSL3-induced ferroptosis, suggesting that this approach could represent a promising therapeutic strategy for *PIK3CA*-mutant CRC [63,64,65,66].

#### 2.5.3. Ferroptosis as an Immunotherapy Enabler

Ferroptosis has been demonstrated to enhance immune checkpoint inhibitor efficacy through multiple mechanisms, functioning as a form of immunogenic cell death (ICD). Ferroptotic tumor cell death causes the release of damage-associated molecular patterns (DAMPs), oxidized phospholipids and proinflammatory cytokines, including interferons and IL-6, which increase tumor antigen presentation and activate dendritic cells and CD8+ T cells. This process, termed “immunoferroptosis”, creates a proinflammatory TME that enhances antitumor immune responses [66,67,68,69,70,71].

CD8+ T cells activated by immunotherapy can directly induce ferroptosis in tumor cells. Mechanistically, interferon-gamma (IFNγ) released from CD8+ T cells downregulates the expression of SLC3A2 and SLC7A11, impairing cystine uptake by tumor cells and promoting lipid peroxidation and ferroptosis. In mouse models, depletion of cystine or cysteine by cyst(e)inase in combination with checkpoint blockade synergistically enhances T-cell-mediated antitumor immunity and induces ferroptosis in tumor cells [69].

Ferroptosis reshapes the TME by regulating intracellular iron levels, lipid metabolism and antioxidant systems, transforming “cold” tumors into “hot” tumors and enhancing immune checkpoint inhibitor efficacy. Ferroptosis induction can modulate myeloid-derived suppressor cells (MDSCs), promote M2-to-M1 macrophage polarization and reduce the number of immunosuppressive regulatory T cells, thereby alleviating immunosuppression in the TME [67,68,72,73]. The combination of ferroptosis induction with immune checkpoint blockade and MDSC depletion may represent an effective approach for reshaping the immune-cold TME of colorectal cancer liver metastases into an immunologically “hot” state capable of supporting durable antitumor immunity [67,68].

#### 2.5.4. Clinical Translation and Combination Strategies

Multiple ferroptosis inducers have shown promise in preclinical and clinical studies of CRC. The dual PI3K/HDAC inhibitor BEBT-908 induces immunogenic ferroptosis and potentiates anti-PD-1 therapy in mice by hyperacetylating p53 and facilitating ferroptotic signaling, leading to the upregulation of MHC class I and the activation of IFNγ signaling via the STAT1 pathway [74]. The ferroptosis inducer RSL3 enhances anti-PD-1 therapy efficacy by activating STAT1 signaling through chaperone-mediated autophagic degradation of SHP2, increasing cancer cell sensitivity to IFNγ and T-cell-mediated cytotoxicity [75].

Advances in nanotechnology have facilitated the precise delivery of ferroptosis inducers or immunomodulators to the TME, enabling targeted therapy that minimizes systemic toxicity [67,76]. Engineered exosomes targeting ferroptosis represent a novel approach to reverse immune checkpoint inhibitor resistance [77]. Additionally, the gut microbiota influences ferroptosis susceptibility in CRC through metabolite production, with short-chain fatty acids sensitizing tumors to ferroptosis, while indole-3-acrylic acid confers resistance through the AHR-ALDH1A3-FSP1/CoQ axis [78].

Finally, an increasingly recognized dimension of metabolic regulation in liver metastasis is the role of tumor-derived extracellular vesicle (EV)-associated microRNAs. Li et al. (2024) reported that breast cancer-derived exosomal miR-9-5p targets *INSIG1*, *INSIG2*, and ATF3 in hepatocytes, upregulating HMGCR and CH25H to drive cholesterol synthesis and 25-hydroxycholesterol production, which polarizes Kupffer cells to facilitate immune evasion and liver metastasis [79]. Importantly, this mechanism may not be specific to breast cancer. A recent study by Cao et al. (2025) revealed that CRC-derived EVs from highly metastatic cells drive hepatic lipid accumulation and premetastatic niche formation, with Kupffer cells as the primary EV recipients, inducing TNFα secretion and further lipid dysregulation [80]. Additionally, *SREBP2*-dependent cholesterol biosynthesis has been shown to be specifically activated in CRC liver metastases, driven by hepatocyte growth factor (HGF) via the c-Met/PI3K/AKT/mTOR axis, and inhibition with betulin or simvastatin suppressed liver metastasis in experimental models [81]. Lipid deposition in CRC liver metastases has also been linked to *YTHDF3*-mediated m6A modification and PPARα degradation [82]. These findings collectively suggest that the cholesterol-rich hepatic microenvironment is critical for vulnerability to CRC liver metastasis and that EV-mediated lipid reprogramming, potentially involving miR-9-5p, warrants direct investigation in EOCRC. Several other miRNAs have been shown to regulate ferroptosis in CRC by targeting key mediators: miR-148a-3p promotes CRC cell ferroptosis by directly targeting *SLC7A11*, leading to GPX4 downregulation, lipid peroxidation, and iron accumulation [83]; miR-15a-3p directly targets GPX4 in CRC, and its suppression reduces sensitivity to the ferroptosis inducer erastin [84]; and miR-509-5p promotes CRC ferroptosis through *SLC7A11* suppression [85]. The incorporation of microRNA-based regulatory mechanisms into the broader framework of ferroptosis and metabolic reprogramming in EOCRC liver metastases represents a productive and currently underdeveloped research direction.

In summary, ferroptosis represents a promising therapeutic strategy for colorectal cancer that can overcome therapy resistance and enhance immunotherapy efficacy, particularly in *PIK3CA*-mutant tumors and immune-cold liver metastases. The combination of ferroptosis inducers with immune checkpoint inhibitors, targeted therapies, and novel delivery systems warrants further clinical investigation to optimize personalized treatment strategies [53,54,67,68].

## 3. Clinical Features of High-Risk EOCRC Patients with Liver Metastases

High-risk patient populations most at risk for the molecular signature associated with EOCRC liver metastases are distinguished by several clinical features. As stated above, younger patients, especially females, more frequently present with primary tumors located in the rectum or left colon. These patients are more likely to have synchronous liver metastases at the time of initial diagnosis and exhibit lymph node involvement, reflecting a more aggressive disease phenotype [23].

A significant subset has a family history of CRC or known hereditary syndromes, particularly Lynch syndrome, which is associated with high MSI due to germline mutations in MMR genes (MLH1, MSH2, MSH6, PMS2) [2,4,86]. In sporadic cases, EOCRC patients often present with poorly differentiated tumors, signet ring cell or mucinous histology and elevated pretreatment carcinoembryonic antigen (CEA) levels, all of which are independent risk factors for liver metastasis [19,23,87].

These populations are characterized by increased rates of *TP53* and *CTNNB1* mutations and lower rates of *APC*, *KRAS* and *BRAF* mutations in MSS tumors, as well as a predominance of consensus molecular subtype 1 (CMS1) in very young patients. These clinical and molecular features collectively identify the high-risk groups most susceptible to EOCRC liver metastases [2,19,86].

Collectively, these clinical features, younger age, female predominance, left-sided tumor location, synchronous metastases, nodal involvement, adverse histology and elevated CEA, define a high-risk phenotype that aligns closely with the molecular alterations characteristic of EOCRC liver metastases.

**Figure 2:**
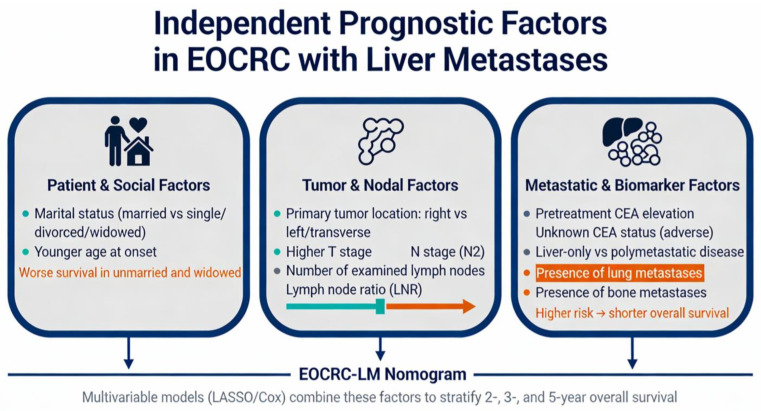
Independent prognostic factors in early-onset colorectal cancer patients with liver metastases. Schematic infographic depicting the main domains integrated into EOCRC-LM nomograms, including patient and social factors (such as marital status), primary tumor and nodal characteristics (location, T and N stage, lymph node evaluation, and lymph node ratio) and metastatic and biomarker features (pretreatment CEA and the presence of lung or bone metastases), all converging into multivariable models that stratify overall survival.

Recent comprehensive nomograms for EOCRC patients with liver metastasis have identified multiple independent prognostic factors that significantly predict overall survival in patients who underwent primary tumor resection combined with chemotherapy. A 2025 study using SEER database data (2010–2015) from 1049 EOCRC-LM patients identified eight independent prognostic factors through LASSO and multivariate Cox regression: marital status, tumor location, T/N stage, CEA level, lymph node count, and bone/lung metastases [88]. The key prognostic domains integrated into the EOCRC-LM nomograms are schematically illustrated in Figure 2.

Marital status has emerged as a significant independent prognostic factor, with unmarried/single and divorced/separated/widowed patients demonstrating significantly worse outcomes than married patients (HR 1.48 and 1.29, respectively) [88]. This finding is consistent with broader CRC literature demonstrating that married patients have better cancer-specific survival than unmarried patients across all stages (time ratio 1.36, 95% CI 1.35–1.37) [88]. The survival benefit correlated with marriage appears to be mediated by earlier diagnosis (accounting for approximately 11% of the effect in CRC patients) and improved treatment receipt, with married patients being more likely to receive definitive therapy (adjusted OR 1.53, 95% CI 1.51–1.56) [89]. The protective effect of marriage is greater in men than in women, and widowed patients represent a particularly vulnerable subgroup with consistently poor outcomes despite often having more favorable clinicopathologic characteristics [90,91] (Figure 2).

Primary tumor location is a well-known prognostic factor in CRC liver metastases. Compared with left-sided tumors, right-sided tumors confer a worse prognosis (HR 1.34 in EOCRC-LM; meta-analysis HR 1.55 for overall survival after resection) [88,91,92,93]. This prognostic impact persists even after adjusting for the KRAS mutational status, although the effect is most pronounced in KRAS wild-type tumors [94]. Right-sided primary tumors are associated with higher rates of RAS mutations (60% vs. 34.9%), positive primary tumor lymph nodes and bilateral hepatic involvement [92]. Notably, transverse colon tumors demonstrate prognostic and predictive features comparable to those of right-sided tumors rather than those of left-sided tumors [92,95] (Figure 2).

TNM stage remains a critical prognostic determinant. A higher T stage and particularly a higher N stage (N2 vs. N0) were significantly associated with worse overall survival (HR for N2: 1.89). Nodal involvement is a critical prognostic determinant, with the presence and extent of lymph node metastases strongly predicting outcomes [88,96,97,98] (Figure 2).

The CEA status is a powerful prognostic biomarker. Elevated pretreatment CEA levels significantly predict worse survival (HR 1.63 for positive, HR 1.49 for unknown) [88]. A meta-analysis of 11,143 patients demonstrated that high prehepatectomy serum CEA levels are correlated with poor overall survival (HR 1.61, 95% CI 1.49–1.75) and recurrence-free survival (HR 1.27, 95% CI 1.11–1.45) [99]. Postoperative CEA levels >5 ng/mL are independent predictors of worse outcomes (HR 2.77 for recurrence-free survival, HR 3.18 for overall survival) [100]. Dynamic CEA changes also have prognostic value, with a CEA increase (CEA-3d higher than both 5 ng/mL and CEA-1 m) showing better discriminatory ability than single CEA measurements [101]. The optimal CEA cutoff value for prognostic stratification has increased in the era of modern chemotherapy, from >5 ng/mL historically to >50–70 ng/mL currently [102].

The extent of lymph node dissection significantly impacts staging accuracy and prognosis. The examination of 17 or fewer regional lymph nodes was associated with worse overall survival (HR 1.32), likely reflecting incomplete staging rather than a true protective effect of more extensive dissection. The AJCC and College of American Pathologists recommend the examination of a minimum of 12 lymph nodes to accurately stage colorectal cancers. The number of negative lymph nodes is an independent prognostic factor, and inadequate lymph node retrieval may result in stage migration and undertreatment. The lymph node ratio (LNR), the ratio of metastatic to examined lymph nodes, has demonstrated superior prognostic value compared with traditional pN staging in multiple studies, with optimal cutoff values of 0.17, 0.41 and 0.69 for prognostic differentiation [88,103,104] (Figure 2).

Metastatic sites significantly impact survival. The presence of bone metastases (HR 2.04) and lung metastases (HR 1.64) significantly worsens survival, indicating that oligometastatic disease localized to the liver confers a better prognosis than polymetastatic disease does. Liver metastasis is associated with a shorter duration of frontline therapy (13 vs. 19.7 months), which is indicative of potential chemotherapy resistance [77,88,97] (Figure 2).

The EOCRC-LM nomogram demonstrated excellent concordance between the predicted and actual survival outcomes, with time-dependent ROC curve analysis revealing AUC values of 0.70, 0.72 and 0.77 for 2-, 3- and 5-year overall survival prediction in the training set, respectively, and comparable AUCs of 0.68, 0.71 and 0.80 in the validation set, respectively [88]. Decision curve analysis demonstrated that these predictive models provide superior clinical applicability compared with traditional TNM staging alone [77,88,97]. Multiple other nomograms for colorectal liver metastases have been developed with similar performance characteristics. A nomogram incorporating 10 clinicopathological factors achieved C-indexes of 0.816, 0.782 and 0.787 for predicting 1-, 3- and 5-year OS, respectively [105]. Dynamic prognostic models incorporating longitudinal laboratory markers (CEA, CA19-9, GGT, RDW, NLR, APRI, and FIB-4) demonstrated enhanced performance, with AUCs of 0.786 at 1 year, 0.748 at 3 years and 0.743 at 5 years for OS [106].

Patients can be stratified into low-, intermediate- and high-risk groups on the basis of nomogram-predicted probabilities, with significant differences in OS between risk strata (log-rank *p* < 0.001) [88]. This stratification enables more precise treatment planning and patient counseling, allowing escalation of therapy intensity in high-risk populations and potential de-escalation in highly favorable-risk patients. Importantly, in the stratified analysis of EOCRC-LM, the prognostic value of age itself may be masked by the underlying biological characteristics of the tumor [88] (Figure 2).

## 4. Therapeutic Strategies Guided by the Molecular Landscape of EOCRC

While standard management of colorectal liver metastases (CRLMs) involves a combination of resection and systemic chemotherapy, the distinct biological behavior of EOCRC necessitates a more tailored approach. EOCRC is frequently characterized by a more aggressive phenotype, including a higher incidence of synchronous metastases and mucinous histology; however, these patients often possess the physiological reserve to tolerate intensive multimodal therapy [23,89]. The emerging treatment paradigm for EOCRC with liver metastases is shifting from age-based aggression to biology-driven precision.

### 4.1. Surgical Aggression and Perioperative Strategy

The clinical phenotype of EOCRC often presents a paradox: patients frequently present with advanced stage disease and adverse features, such as lymph node metastasis (57.4% vs. 48.2%) and bilobar liver involvement, yet survival outcomes comparable to those of older cohorts when matched for stage [23,90]. This finding supports a strategy of aggressive surgical intervention. However, the decision to resect should increasingly be informed by molecular risk rather than anatomical resectability alone. While perioperative chemotherapy (FOLFOX/CAPEOX) remains the standard for testing tumor biology and clearing micrometastases [92,95], the molecular identity of the tumor must guide the surgical window. For high-risk EOCRC patients—who are more likely to harbor aggressive molecular subtypes—a “liver-first” or simultaneous resection approach is often justified to address the burden of disease, provided that the tumor biology is responsive to neoadjuvant agents. The comparable recurrence-free survival rates between early- and late-onset cohorts (24.2% vs. 24.8%) despite the aggressive presentation of EOCRC suggest that when the distinct biology of EOCRC is managed with maximal surgical effort, the “aggressive” phenotype can be neutralized [23].

### 4.2. Exploiting the MSI-H/dMMR Landscape in EOCRC

A critical difference in EOCRC is the increased prevalence of sporadic and hereditary (Lynch syndrome) MSI-H/dMMR. Unlike the general CRC population, where MSI-H is rare in metastatic disease, the enrichment of this subtype in young patients mandates universal germline and somatic testing to unlock immunotherapy options. For the subset of EOCRC patients with dMMR/MSI-H liver metastases, immune checkpoint inhibition has displaced chemotherapy as the standard of care. The KEYNOTE-177 trial data are particularly relevant for younger patients, offering not only improved survival (HR 0.60 for PFS) but also a durable response that acts as a bridge to potentially curative liver resection in previously unresectable patients [96,97]. In the context of EOCRC, where long-term quality of life is paramount, the reduced toxicity profile of pembrolizumab compared with that of cytotoxic chemotherapy is a significant consideration.

### 4.3. Overcoming Resistance in MSS EOCRC

The majority of EOCRC cases are MSS, presenting a profound therapeutic challenge that is compounded by the immune-cold liver metastatic microenvironment characterized in detail in Section 2.4. This section focuses specifically on the therapeutic strategies designed to address this suppressive architecture within the EOCRC molecular context [32,101]. To address this, novel combinations specifically target the immunosuppressive architecture common in young, fit patients:

(1) Reversing immune exclusion (LIGHT/TNFSF14): Preclinical data suggest that inducing the expression of LIGHT (TNFSF14) can remodel the “cold” TME of liver metastases. By promoting the formation of tertiary lymphoid structures and recruiting T cells, this approach, combined with anti-CTLA-4, aims to overcome the specific mechanisms of hepatic immune tolerance [88,103,104,105].
(2) VEGF and TKI Combinations: Recognizing that angiogenesis pathways often coregulate immune suppression, combinations such as regorafenib plus sintilimab have shown promise in MSS mCRC. This is particularly relevant for EOCRC patients, who are often RAS/BRAF wild-type, a subgroup that demonstrated significantly prolonged survival (median OS 23.3 months) with this regimen [107].

### 4.4. Metabolic and Molecular Precision: The PI3K/mTOR Axis

Emerging research into the metabolic deregulation of EOCRC offers new therapeutic avenues beyond standard cytotoxic agents. *PIK3CA* mutations, while found across age groups, present a specific vulnerability in the context of lipid metabolism, which is often altered in EOCRC. The hyperactivated PI3K/AKT/mTOR pathway normally protects cancer cells from ferroptosis (iron-dependent cell death) by upregulating lipid-protective enzymes such as SCD1. Therapeutic strategies that disrupt this protective mechanism are currently being defined.

Specifically, the combination of aspirin and ferroptosis inducers, such as RSL3, targets this axis. Aspirin-mediated mTOR inhibition synergizes with RSL3-induced ferroptosis, suggesting that this approach could represent a promising therapeutic strategy for *PIK3CA*-mutant CRC [46,47,48]. This is not merely an additive effect but a mechanistic synergy: aspirin suppresses the mTOR/SREBP-1/SCD1 axis, stripping the tumor from its metabolic defenses against ferroptosis. For young patients with *PIK3CA*-mutant disease, this represents a rational, biologically driven addition to standard regimens, moving treatment from generic protocols to precision metabolic intervention.

However, current evidence strongly suggests that pharmacological mTOR suppression can promote the generation and survival of dormant cancer cells, including those in CRC models. This represents a paradoxical consequence of mTOR inhibition: while it suppresses tumor proliferation, it simultaneously facilitates the emergence of drug-tolerant, quiescent persister populations that may seed future recurrence. Specifically, a genome-wide CRISPR screen in pancreatic cancer cells identified the mTOR pathway as a prominent determinant of chemosensitivity. Pharmacological mTOR suppression across cancer cells of diverse tissue origins led to the persistence of a reversibly resistant population displaying a senescence-like phenotype. Importantly, mTOR inhibition does not induce senescence per se but promotes the survival of senescent cells through autophagy upregulation and G2/M cell cycle arrest [107]. Pretreatment with pharmacological mTOR inhibitors synergizes with low cell density to induce self-sustained quiescence (SSQ) in a Beclin-1-dependent manner. The cells in SSQ form small indolent tumors that eventually transition to rapid growth, and up to 40% of clonogenic cancer cells from resected tumors can enter this state [108]. Specifically, in CRC, the relationship between mTOR and dormancy operates through several pathways. Dormant CRC cells exhibit altered signaling through the Hippo/YAP, NANOG, HIF-1α, Notch, and ERK/p38/MAPK pathways, with the AKT/mTOR axis playing a central regulatory role [109,110]. PI3K/mTOR blockade in CRC cells induces protumorigenic senescence mediated by stress kinase p38 activation, with senescent cells secreting cytokines that paradoxically increase the migration and invasion of neighboring CRC cells [111]. Chemotherapy-induced CRC cell death triggers ATP release and P2X4-mediated mTOR-dependent prosurvival signaling in neighboring cells, creating a new dependency that can be therapeutically exploited [112]. Notably, mTOR inhibition in CRC also affects cancer stem-like cells (CSCs). While rapamycin and PP242 decrease sphere formation and ALDH activity in CRC cells, mTOR inhibitors can simultaneously suppress the stimulation of stem-like cells by chemotherapy, a double-edged effect where reduced stemness may coexist with increased dormancy [113]. These findings collectively underscore that mTOR inhibition in oncology must be considered within the framework of dormancy biology, particularly in CRC, where late recurrence from dormant disseminated cells remains a major clinical challenge.

## 5. Conclusions, Future Directions and Research Priorities

Early-onset colorectal cancer with liver metastases exhibits a particularly aggressive biological profile characterized by distinct molecular alterations, increased genomic instability and a counterintuitive immune-cold phenotype despite heightened immunogenicity. Recent advances in elucidating the molecular landscape, encompassing CNV burden, ferroptosis sensitivity and immune microenvironment composition, offer tangible opportunities for precision therapeutic intervention. The inadequacy of conventional immunotherapy in MSS CRC liver metastases, particularly in the EOCRC context, makes the exploration of combination strategies that leverage ferroptosis induction, dendritic cell recruitment, MDSC depletion and checkpoint blockade not only rational but also urgent.

The development of comprehensive prognostic nomograms has refined risk stratification and personalized treatment planning in this population. Translating these mechanistic insights into clinical benefit will require collaborative, multicenter research, biomarker-driven trial designs and a sustained commitment to understanding the distinctive biology of EOCRC. Crucially, this research must extend to incorporating emerging evidence on mTOR-driven cancer cell dormancy, a mechanism by which EOCRC cells may evade both chemotherapy and targeted agents by entering a reversible diapause-like state, into the design of future therapeutic strategies.

Priority areas for future mechanistic research include the elucidation of the pathways driving the progenitor-like phenotype and cancer stem cell enrichment in metastatic EOCRC, the mechanisms governing the transition from immune-cold to immune-hot microenvironments, and a thorough characterization of ferroptosis resistance in EOCRC. Integration of single-cell transcriptomics with functional assays will be critical for identifying the cellular populations that drive metastatic aggressiveness.

Future clinical trials should prioritize phase II/III studies of innovative immunotherapy combinations, including the LIGHT-ICI, FLT3L-ICI and ferroptosis-ICI regimens, which specifically target EOCRC-derived liver metastasis populations. Trial designs should select participants on the basis of tumor genomics and immune signatures rather than age alone. The examination of adjuvant ferroptosis-based strategies aimed at preventing liver metastases in early-stage EOCRC and comparative effectiveness studies of resection-based versus chemotherapy-only approaches in EOCRC-LM are also warranted.

In addition to current paradigms, emerging technologies represent a particularly promising frontier for EOCRC liver metastasis research. Liquid biopsy, through circulating tumor DNA and circulating tumor cells, offers a real-time, minimally invasive window into tumor evolution, treatment response and early detection of metastatic recurrence, which is especially valuable in the young patient population where repeated tissue sampling is poorly tolerated. Patient-derived organoids from EOCRC liver metastases provide physiologically relevant models that capture the molecular heterogeneity of individual tumors and enable personalized drug sensitivity testing prior to clinical decision-making. Spatial transcriptomics can be used to decipher the complex architecture of the EOCRC liver metastatic niche, mapping the precise spatial relationships between immune, stromal and tumor cell populations that underpin the immune-cold phenotype and resistance to combination immunotherapy. Systematic incorporation of these technologies into EOCRC-specific research programs will substantially accelerate the translation of molecular insights into clinical benefit.

## Data Availability

No new data were created or analyzed in this study. Data sharing is not applicable to this article.
